# Supplementary material for: A molecular mechanism to diversify Ca2+ signaling downstream of Gs protein-coupled receptors
Source: Nat Commun. 2024 Sep 3;15:7684. doi: 10.1038/s41467-024-51991-6 (PMC11372221; doi:10.1038/s41467-024-51991-6)

Supplementary Information

A molecular mechanism to diversify Ca^{2+} signaling downstream of Gs protein-coupled receptors

Julian Brands[1,2,15], Sergi Bravo[1,15], Lars Jürgenliemke[1,3,15], Lukas Grätz[4], Hannes Schihada[5], Fabian Frechen[6], Judith Alenfelder[1], Cy Pfeil[1,2,12], Paul Georg Ohse[1], Suzune Hiratsuka[7], Kouki Kawakami[7,13], Luna C. Schmacke[5], Nina Heycke[1], Asuka Inoue[7,8], Gabriele König[9], Alexander Pfeifer[10], Dagmar Wachten[6], Gunnar Schulte[4], Torsten Steinmetzer[5], Val J. Watts[11], Jesús Gomeza[1,16], Katharina Simon[1,14,16], Evi Kostenis[1,16,*]

1) Molecular, Cellular and Pharmacobiology Section, Institute for Pharmaceutical Biology, University of Bonn, Bonn, Germany
2) Research Training Group 1873, University of Bonn, Bonn, Germany
3) Research Training Group 2873, University of Bonn, Bonn, Germany
4) Department of Physiology and Pharmacology, Karolinska Institutet, Stockholm, Sweden
5) Department of Pharmaceutical Chemistry, Philipps-University Marburg, Marburg, Germany
6) Institute of Innate Immunity, Medical Faculty, University of Bonn, Bonn, Germany
7) Graduate School of Pharmaceutical Sciences, Tohoku University, Sendai 980-8578, Japan
8) Graduate School of Pharmaceutical Sciences, Kyoto University, Kyoto 606-8501, Japan
9) Institute for Pharmaceutical Biology, University of Bonn, Bonn, Germany
10) Institute of Pharmacology and Toxicology, University Hospital, University of Bonn, Bonn, Germany
11) Department of Medicinal Chemistry and Molecular Pharmacology, Purdue Institute of Drug Discovery, Purdue University, West Lafayette, Indiana
12) Present address: Amsterdam Institute for Molecular and Life Sciences (AIMMS), Division of Medicinal Chemistry, Faculty of Science, Vrije Universiteit Amsterdam, Amsterdam, Netherlands
13) Present address: Komaba Institute for Science, The University of Tokyo, Meguro, Tokyo 153-8505, Japan
14) Present address: Department of Pharmaceutical and Pharmacological Sciences, University of Padova, 35131 Padova, Italy
15) These authors contributed equally: Julian Brands, Sergi Bravo, Lars Jürgenliemke
16) Shared senior authors

* Correspondence to Evi Kostenis (kostenis@uni-bonn.de)

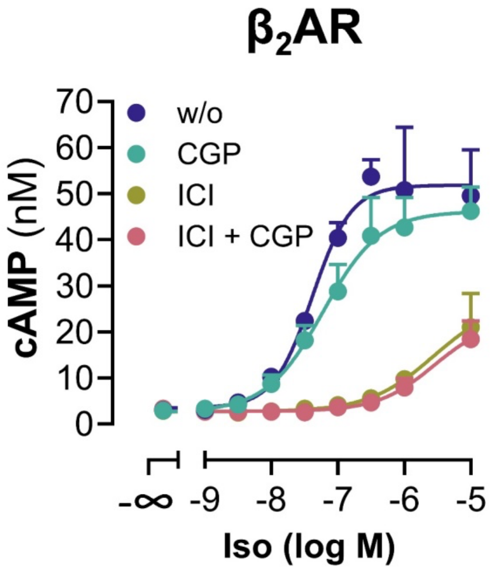

Supplementary Fig. 1: Isoproterenol stimulates cAMP accumulation in HEK293 cells via endogenous β$_2$ARs. Addition of isoproterenol (Iso) to naïve HEK293 cells caused an increase of the intracellular second messenger cAMP. Treatment of cells with the β$_2$AR-specific inhibitor ICI-118,551 (ICI) (100 nM) blocked the Iso-mediated cAMP increase, whereas CGP-20712A (300 nM), a β$_1$AR-specific inhibitor, hardly impacted cAMP formation when applied alone or in combination with ICI. Data are means + SD of n=2 independent experiments, each performed in triplicate. Source data are provided as a Source Data file.

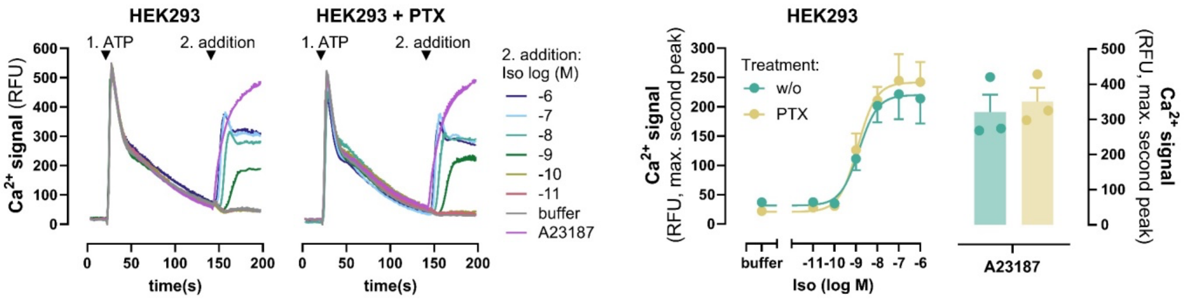

Supplementary Fig. 2: Iso-triggered β_2AR-Ca^{2+} after Gq priming is not diminished by PTX pretreatment. HEK293 cells were primed with 100 µM ATP at t = 20 s, followed by a second addition at t = 140 s of either Iso or Calcium ionophore A23187 in the absence or presence of PTX. Shown are representative traces and concentration effect curves derived from the maximum calcium response of the second addition of Iso on β_2AR, as well as bar chart quantification of A23187 (5 µM) after ATP priming. Where indicated, cells were pretreated overnight (16 h) with 100 ng/ml of PTX. Representative traces are mean + SD, averaged data are mean + SEM of n = 3 biologically independent experiments, each performed in duplicate. Source data are provided as a Source Data file.

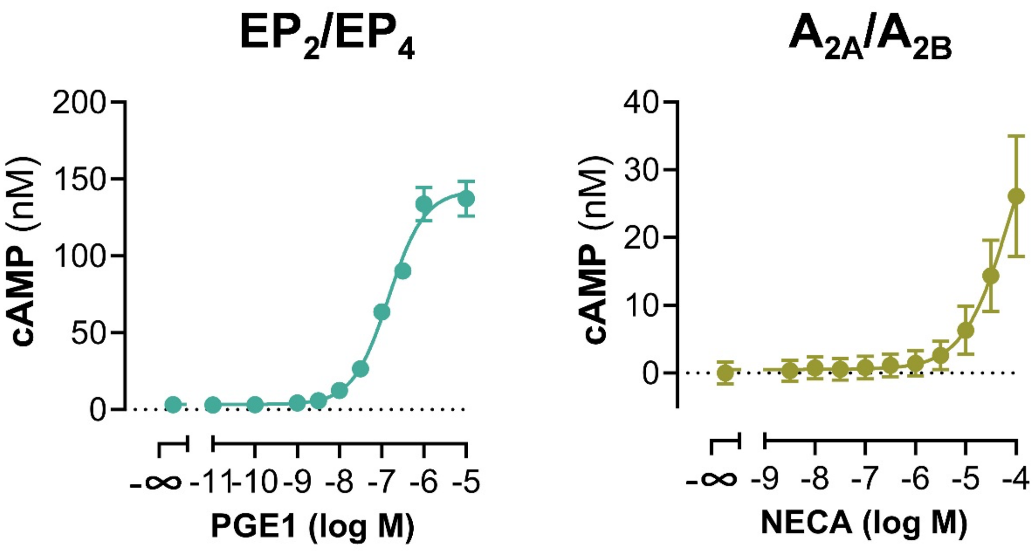

Supplementary Fig. 3: Endogenous Gs-coupled prostanoid EP_2/EP_4 and adenosine A_{2A}/A_{2B} receptors elevate intracellular cAMP after ligand stimulation. Addition of prostaglandin E_1 (PGE_1) to activate endogenous EP_2/EP_4 or of NECA to stimulate endogenous A_{2A}/A_{2B} receptors, respectively, in naïve HEK293 cells caused detectable increases in the intracellular cAMP concentration. Cells were pretreated with 100 ng/ml PTX to produce inactivation of Gi/o proteins. Data are means ± SEM of n = 4 independent experiments, each performed in triplicate. Source data are provided as a Source Data file.

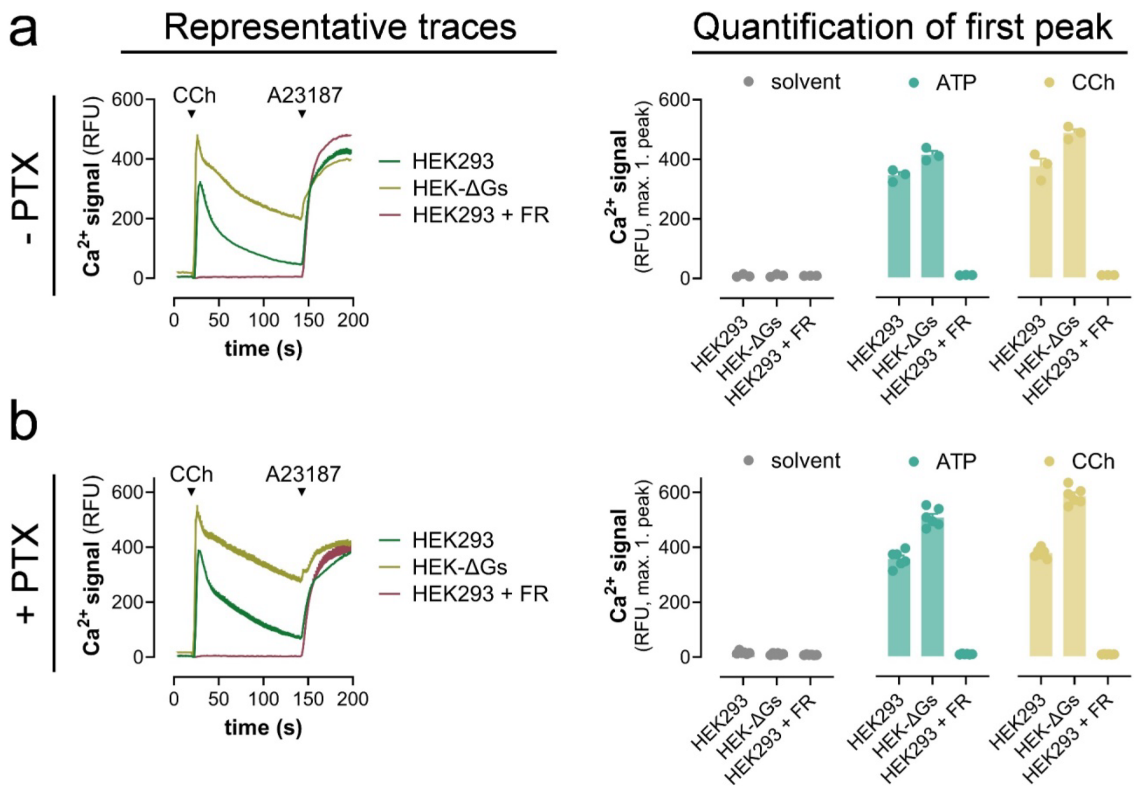

Supplementary Fig. 4: FR pretreatment but not Gα$_s$ deletion abolishes the Gq-mediated first Ca^{2+} peak. **a**, **b** Representative Ca^{2+} traces evoked by 100 µM CCh at t = 20 s and followed by a second addition of 5 µM calcium ionophore A23187 at t = 140 s in HEK293, HEK-ΔGs, and HEK293 cells pretreated with 1 µM of the Gq inhibitor FR. The right panels show the quantification of the first addition as maximum Ca^{2+} amplitudes for either solvent, 100 µM ATP, or 100 µM CCh. Note that representative traces for 100 µM ATP are already contained in the main Figure 1. Cells in (**b**) were treated overnight with 100 ng/ml PTX to inactivate Gi/o. Data are means + SEM of n = 3 independent experiments (**a**) and n = 6 independent experiments (**b**), each performed in duplicate. Source data are provided as a Source Data file.

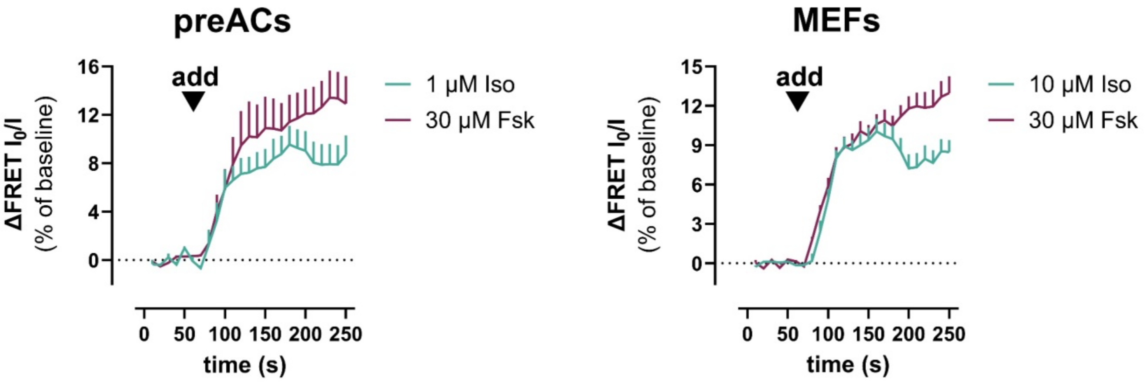

Supplementary Fig. 5: Iso promotes cAMP formation in preACs and MEFs within the Ca^{2+} detection window. FRET recordings of cAMP dynamics in preACs and MEF single cells after addition of either Iso or Fsk, obtained with the pcDNA3.1-mICNDB-FRET sensor[1]; see also Fig. 5e for cartoon illustration of the sensor principle. FRET data for preACs are means + SEM of n = 6 (Iso) and n = 4 (Fsk) cells; FRET data for MEFs are means + SEM of n = 17 (Iso) and n = 11 (Fsk) cells. Source data are provided as a Source Data file.

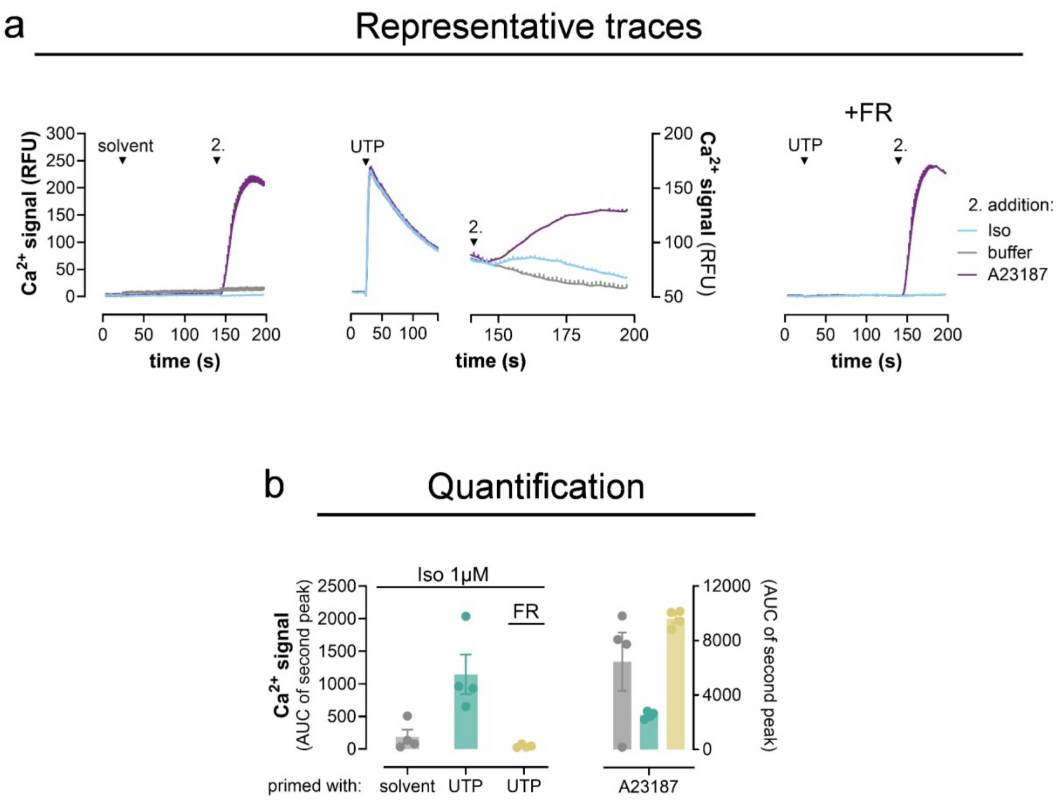

Supplementary Fig. 6: UTP priming is mandatory to elicit Gs-calcium in mouse embryonic fibroblasts (MEFs). Representative calcium recordings (**a**) and their quantification (**b**) obtained in mouse embryonic fibroblasts (MEFs) following a two-step addition protocol. At t = 20 s, cells were primed with solvent or 100 µM UTP (**a**), followed by a second addition at t = 140 s of the βAR stimulus Iso in the absence and presence of 1 µM FR. **b** Bar chart quantification of the data in (**a**) including calcium ionophore A23187 (5 µM) as control. Representative recordings are mean + SEM, bar graphs are mean ± SEM of n = 4 biologically independent experiments, each performed in duplicate. Cells were PTX-pretreated (100 ng/ml, 16 h) to silence any potential Gi/o input to the Ca^{2+} recordings. Source data are provided as a Source Data file.

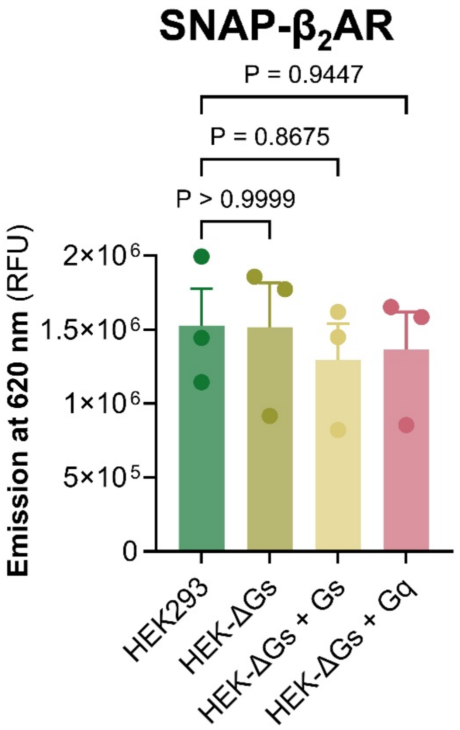

Supplementary Fig. 7: The cellular abundance of β_2ARs is comparable across cell lines and transfection conditions. N-terminally SNAP-tagged β_2AR was labeled with SNAP-Lumi4®-Tb reagent followed by detection of fluorescence intensity (relative fluorescence units, RFU) at 620 nM. The data are plotted as mean + SEM of n = 3 biologically independent experiments. Statistical significance was determined using a one-way ANOVA with Dunnett´s post-hoc analysis. Source data are provided as a Source Data file.

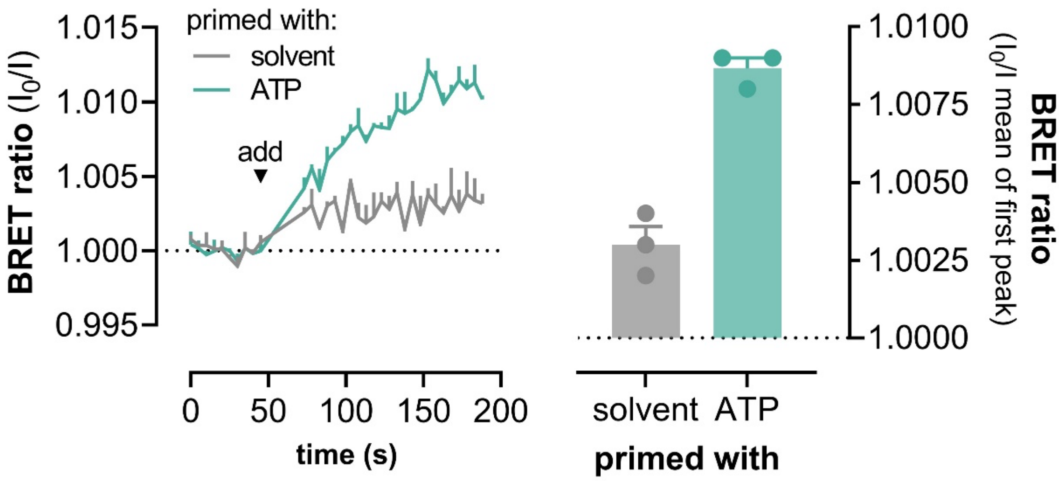

Supplementary Fig. 8: IP_3 is formed during the priming phase within the Ca^{2+} detection window. Real-time BRET recording of intracellular IP_3 formation in response to 100 µM ATP or solvent in HEK293 cells transfected to express the IP_3-BRET sensor[2]; see also Fig. 6c for cartoon illustration of the sensor principle. ATP addition to cells resulted in an immediate increase in intracellular IP_3 levels. Real-time BRET recordings show mean + SEM, the bar chart represents quantification of data as mean + SEM for n = 3 independent experiments, each performed in quadruplicate. Source data are provided as a Source Data file.

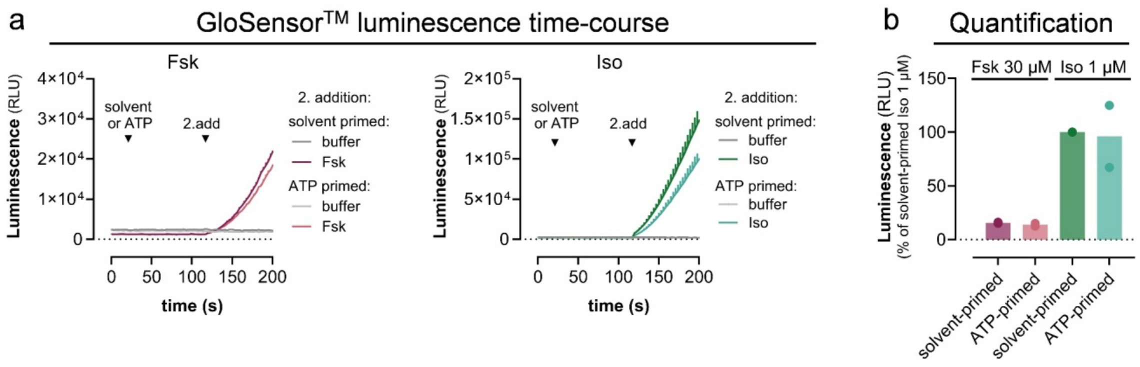

Supplementary Fig. 9: Fsk measurably elevates cAMP within the Ca^{2+} detection window. a, b HEK293 cells were transiently transfected to express the cAMP-GloSensor^{TM} biosensor to resolve the initial kinetic phase of cAMP formation in real-time. **a** Similar to the Ca^{2+} mobilization assays with two consecutive additions, either solvent or 100 µM ATP were added at t = 20 s, followed by the second addition at t = 120 s of either solvent, 30 µM Fsk, or 1 µM Iso as reference stimulus. Fsk-promoted cAMP formation is readily detectable after ligand addition, is independent of the Gq prestimulus and - with respect to Iso - slower in onset and lower in magnitude. **b** Quantification of the peak response maxima in (**a**) for n = 2 independent experiments and expressed as percentage of the 1 µM Iso response obtained with solvent as primer in each individual experiment. The representative GloSensor^{TM} luminescence time-course is the mean + SEM of a triplicate measurement. RLU, relative light units. Source data are provided as a Source Data file.

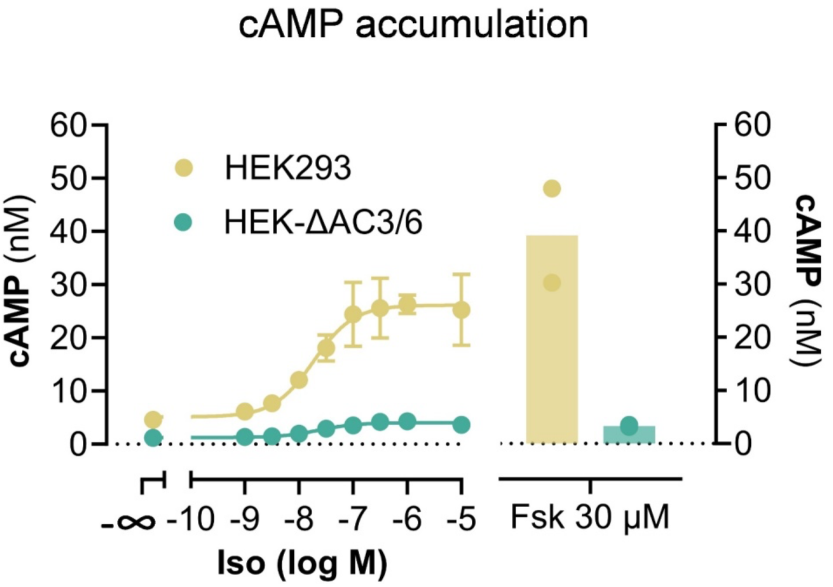

Supplementary Fig. 10: Genetic deletion of adenylyl cyclase (AC) isoforms 3 and 6 in HEK293 cells strongly diminishes intracellular cAMP formation. Cyclic AMP production stimulated with the indicated concentrations of Iso and Fsk in parental HEK293 and HEK-ΔAC3/6 KO cells with targeted deletion of the alleles coding for the AC isoforms 3 and 6. Cytosolic cAMP levels were significantly lower in the HEK-ΔAC3/6 background as compared to the parental cell line, while Iso log EC_{50} values were essentially unaltered (HEK293: Iso pEC_{50}= 7.7 ± 0.3; HEK-ΔAC3/6 KO: Iso pEC_{50}= 7.6 ± 0.5). Concentration-effect curves are means ± SD of n = 2 independent experiments performed in triplicate. Source data are provided as a Source Data file.

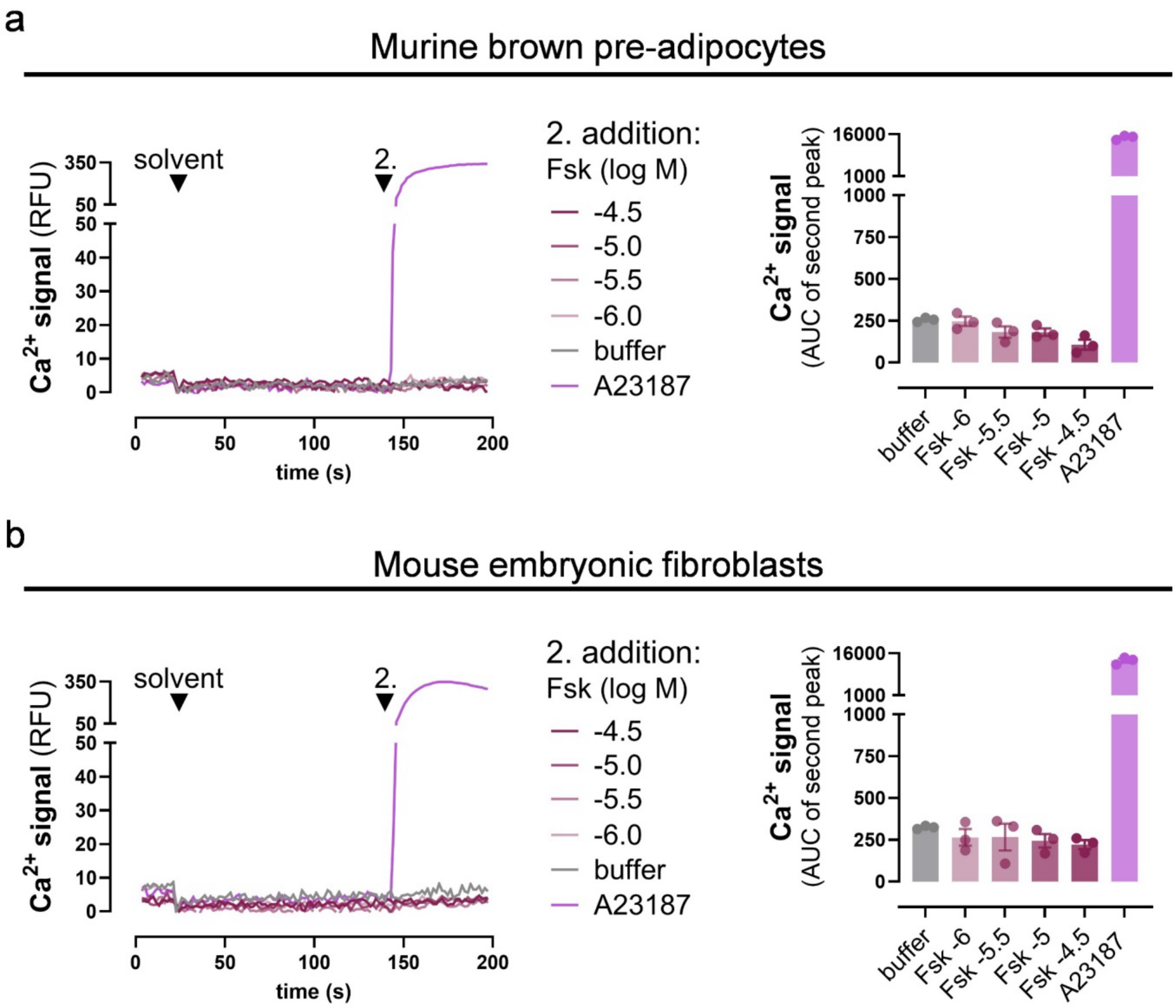

Supplementary Fig. 11: Fsk does not mobilize detectable Ca^{2+} after solvent priming in preACs and MEFs. a, b Representative calcium recordings and their quantification obtained in pre-ACs (**a**) or mouse embryonic fibroblasts (MEFs) (**b**) following a two-step addition protocol. At t = 20 s, cells were treated with solvent, followed by a second addition at t = 140 s of fsk. Bar chart quantification of the data in (**a** and **b**) plotted as area under the curve (AUC) including calcium ionophore A23187 (5 µM) as control. Representative recordings are mean + SEM, bar graphs are mean ± SEM of n = 3 biologically independent experiments, each performed in duplicate. Cells were PTX-pretreated (100 ng/ml, 16 h) to silence any potential Gi/o input to the Ca^{2+} recordings. Source data are provided as a Source Data file.

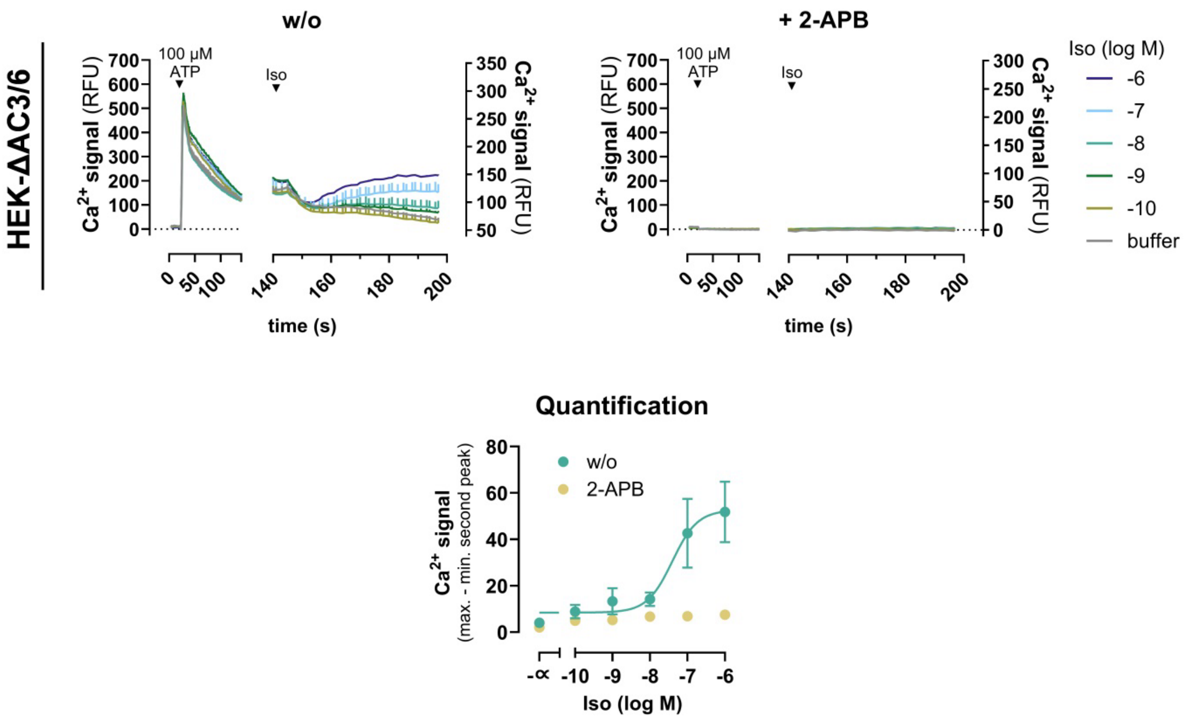

Supplementary Fig. 12: 2-APB eliminates Iso-mediated β_2AR-Ca^{2+} after Gq priming in ΔAC3/6 cells. Calcium mobilization in ΔAC3/6 cells following the two consecutive addition protocol. At t = 20 s, the Gq stimulus ATP 100 µM was added, followed by a second addition at t = 140 s of Iso. Data show representative Iso-induced Ca^{2+} traces and their quantification as concentration-effect-curves in the absence or presence of 50 µM of the IP_3R antagonist 2-APB after ATP priming. Real-time Ca^{2+} recordings are mean values + SEM of technical duplicates, concentration-effect curves are mean values ± SEM of n = 4 (w/o 2-APB) and n = 3 (with 2-APB) independent biological experiments. Source data are provided as a Source Data file.

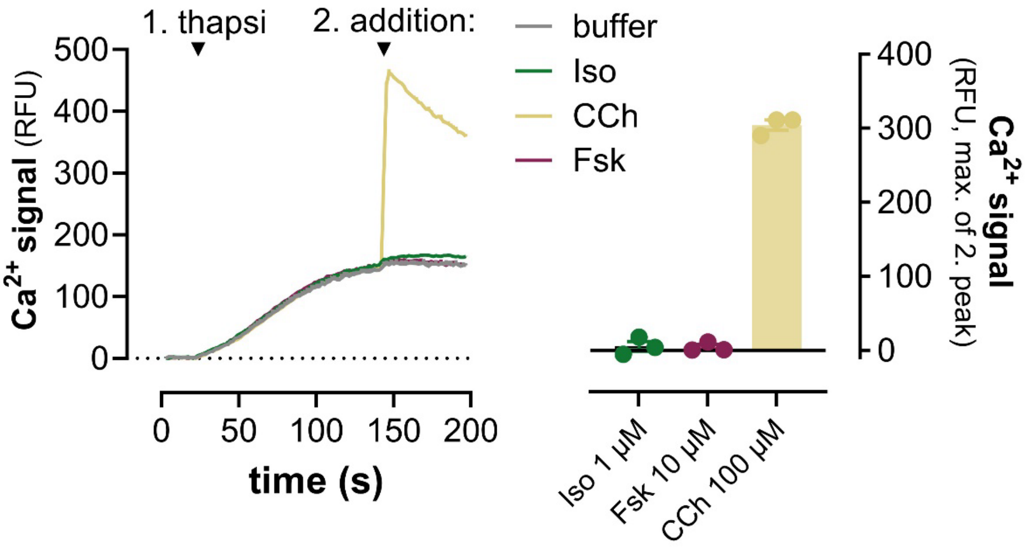

Supplementary Fig. 13: Gq priming but not the mere elevation of cytosolic Ca^{2+} enables Gs-Ca^{2+}. HEK293 cells were first stimulated with 1 µM SERCA inhibitor thapsigargin (thapsi) to increase cytosolic Ca^{2+}, followed by a second addition of 1 µM Iso, 30 µM Fsk, 100 µM CCh or buffer. Shown are real-time Ca^{2+} fluorescence recordings of one representative experiment as mean + SEM and the bar chart quantification displaying the means ± SEM of n = 3 independent experiments, each performed in duplicate. RFU, relative fluorescence units. Source data are provided as a Source Data file.

***PLCB1* (WT)**

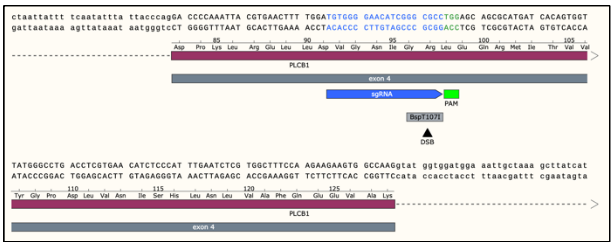

***PLCB1* (allele 1)**

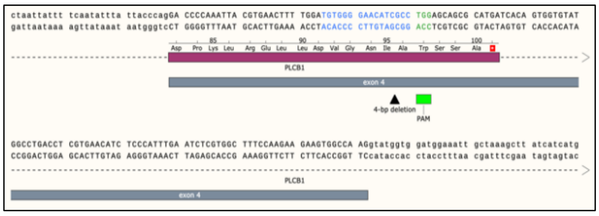

***PLCB1* (allele 2)**

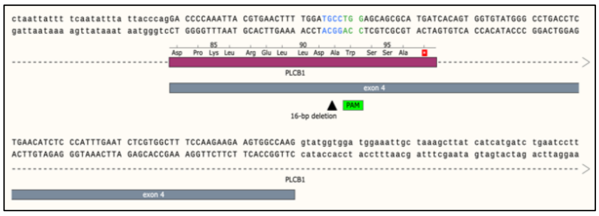

***PLCB2* (WT)**

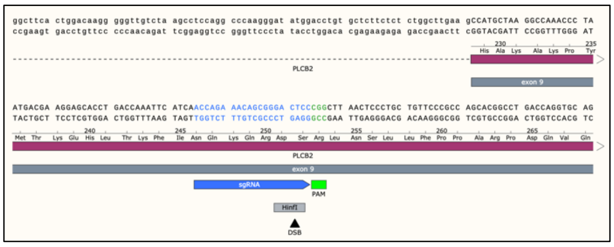

***PLCB2* (allele 1)**

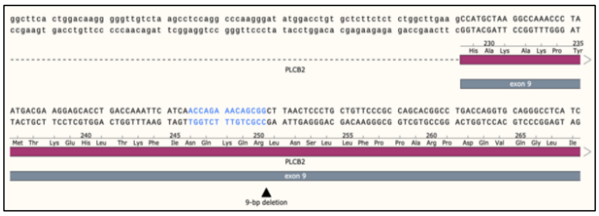

***PLCB2* (allele 2)**

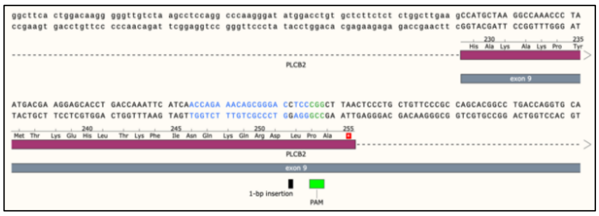

PLCB3 (WT)

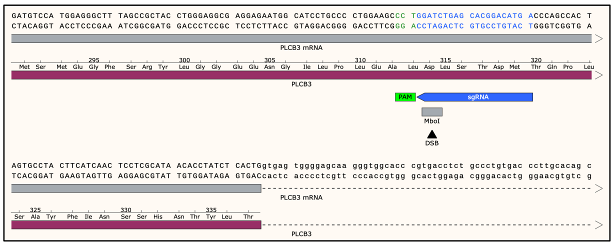

PLCB3 (allele 1)

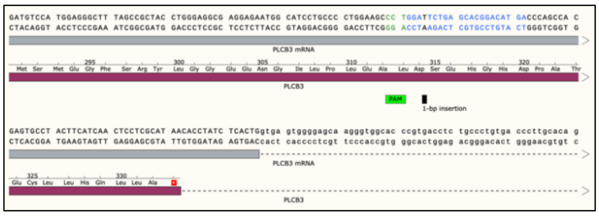

PLCB3 (allele 2)

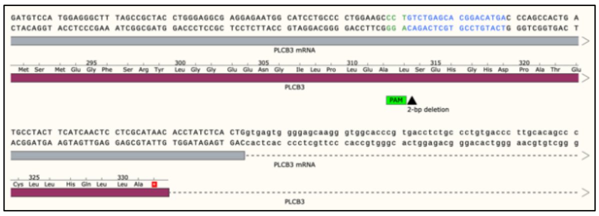

PLCB3 (allele 3)

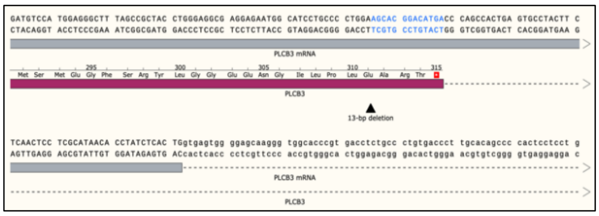

***PLCB4* (WT)**

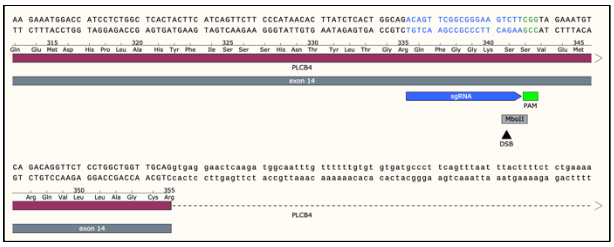

***PLCB4* (allele 1)**

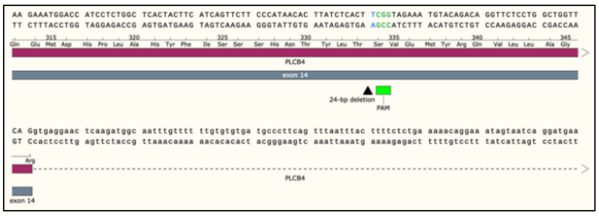

Supplementary Fig. 14: Selected sgRNA and the resulting mutant sequences for each of the PLCβ isoforms. In the panels labeled as WT, *PLCB*-targeting sgRNA sequences, as well as the PAM sequences and the corresponding restriction enzyme for genotype screening, are shown. In the mutant-allele panels, the new sequences of each *PLCB* gene are shown along with the types of mutations (deletion or insertion). DSB, a site of double-stranded break by the *Streptococcus pyogenes* Cas9 (SpCas9) nuclease. The figure panels were generated by the SnapGene software. Note that the established cell line, while harboring the WT sequence in the *PLCB4* gene and an in-frame mutation in the *PLCB2* gene, is unresponsive to Gq-GPCR stimulation and allows investigation of Ca^{2+} signaling of each individual PLCβ isoform after re-expression; see also Supplementary figures 15 and 16).

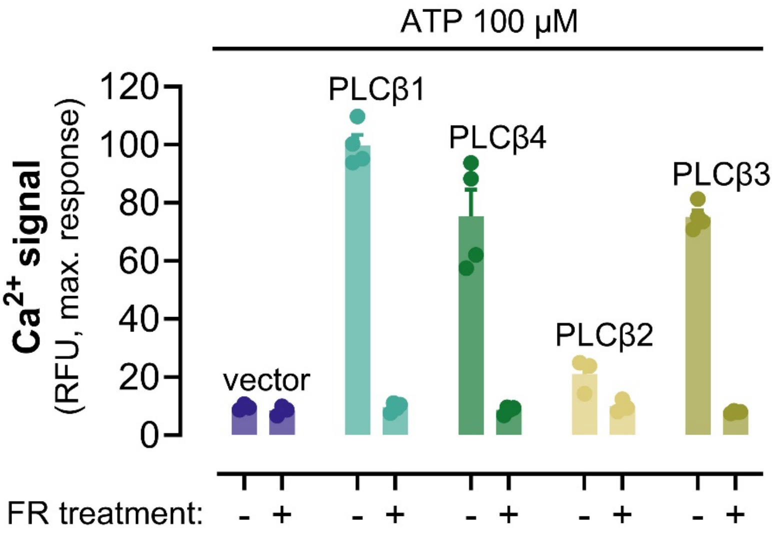

Supplementary Fig. 15: All PLCβ1-4 isoforms maintain their natural regulation by Gq/11 after re-expression in HEK-Δ$_f$PLCβ1-4 cells. HEK-Δ$_f$PLCβ1-4 cells were transfected with each of the individual PLCβ isoforms or empty pcDNA3.1 expression vector and stimulated with ATP as Gq stimulus in the absence or presence of the Gq inhibitor FR. Ca^{2+} responses were undetectable in HEK-Δ$_f$PLCβ1-4 cells, re-emerged with expression of each individual PLCβ isoform, and were eliminated upon pretreatment with 1 µM of the Gq inhibitor FR. Data are mean values + SEM (vector: *n* = 3; PLCβ1 and β4 : *n* = 4; PLCβ2 and β3: *n* = 3), each performed in technical duplicate. Source data are provided as a Source Data file.

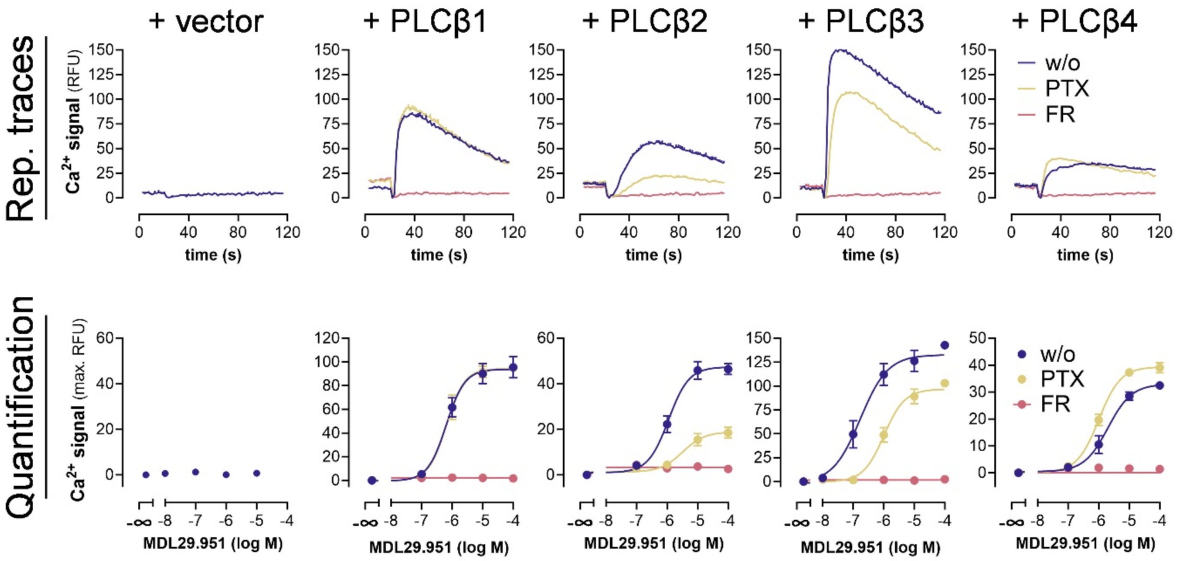

Supplementary Fig. 16: Re-expression of PLCβ1-4 isoforms in HEK-Δ_fPLCβ1-4 mutant cells re-establishes their natural regulation by both Gq/11 and/or Gi-liberated Gβγ subunits. HEK-Δ_fPLCβ1-4 cells do not promote detectable Ca^{2+} in response to a dual Gq/Gi-GPCR stimulus, well-established to liberate intracellular Ca^{2+} in a PTX- and FR-dependent manner (Pfeil et al., 2020). Here, we utilized the Gi/q-sensitive rat ortholog of GPR17 (rGPR17) and its small molecule agonist MDL29.951 to probe natural regulation of PLCβ1-4 by Gq and Gi-Gβγ. Ligand-promoted Ca^{2+} signals in HEK-Δ_fPLCβ1-4 cells transfected to express rGPR17 were strictly dependent on re-expression of each individual PLCβ isoform and showed the isoform-expected sensitivity to pretreatment with the G protein signaling inhibitors FR (no cytosolic Ca^{2+} detectable) and PTX (cytosolic Ca^{2+} increase diminished only after re-expression of Gβγ-regulated PLCβ2 and PLCβ3). Ligands and pathway inhibitors were used at a final concentration of 100 µM (MDL29.951, rep. traces), 1 µM (FR), and 100 ng/ mL (PTX). Concentration-effect curves for peak Ca^{2+} responses are the mean values ± SEM of n = 3 biological replicates, each performed in technical duplicate. Source data are provided as a Source Data file.

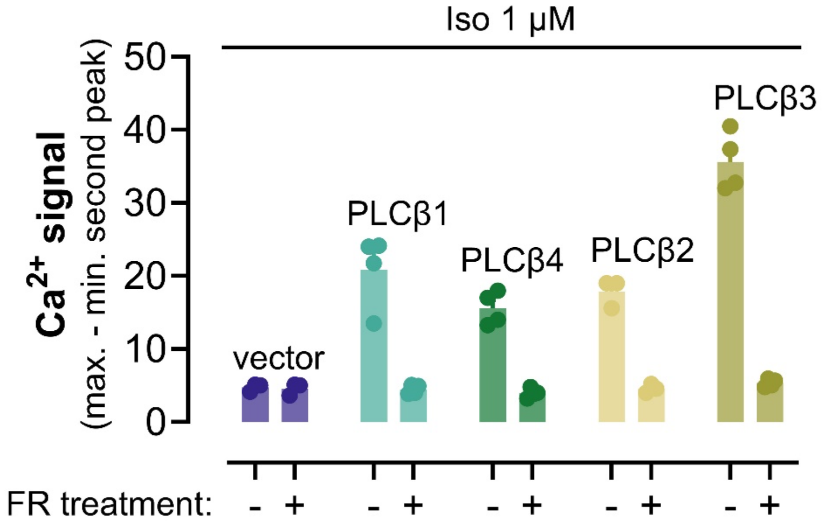

Supplementary Fig. 17: Gs-mediated calcium is Gq-dependent across all four PLCβ isoforms. HEK-Δ_fPLCβ1-4 cells were transfected to express each individual PLCβ isoform and stimulated with 100 µM ATP as a Gq pre-stimulus, followed by the addition of Iso. All cells with PLCβ re-expression empowered Gs calcium after Gq priming, while treatment with 1 µM FR abolished this Gs-mediated response. Data are mean values + SEM (vector: *n* = 3; PLCβ1 and β4: *n* = 4; PLCβ2 and β3: *n* = 3), each performed in technical duplicate. Source data are provided as a Source Data file.

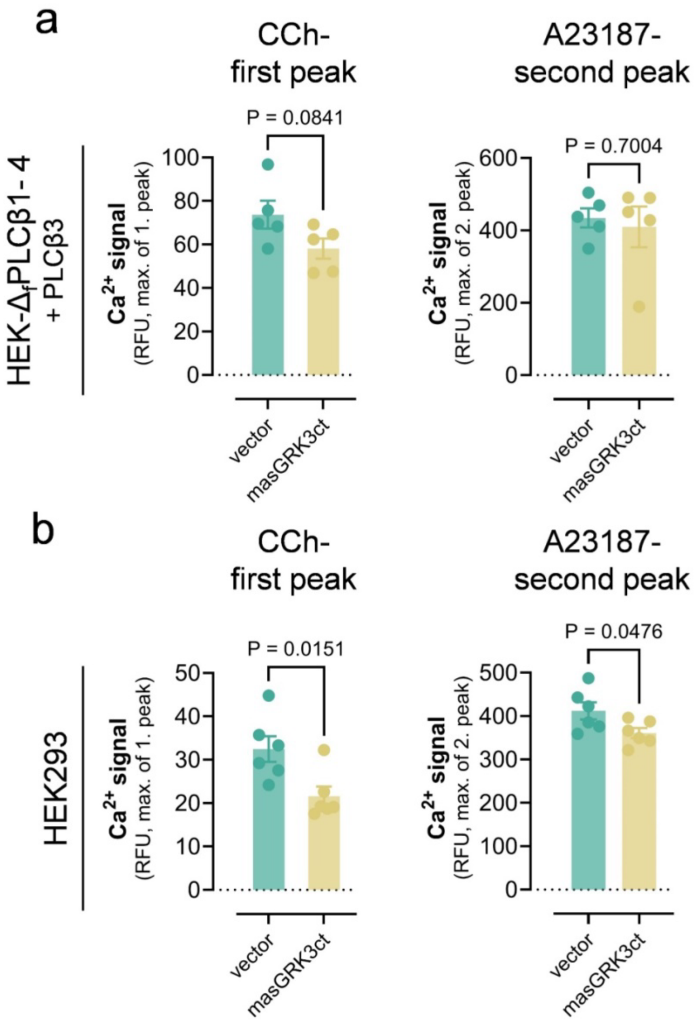

Supplementary Fig. 18: Effect of Gβγ scavenger masGRK3ct on Ca^{2+} transients induced by CCh and the calcium ionophore A23187. Cytosolic Ca^{2+} mobilization measurements in HEK-Δ_fPLCβ1-4 (**a**) or HEK293-wt cells (**b**) transfected with or without the Gβγ scavenger masGRK3ct and stimulated with either CCh or A23187. Bar chart quantifications display the mean peak responses of Ca^{2+} signals for CCh and A23187 in the presence or absence of co-expressed masGRK3ct obtained in n = 5 (**a**) or n = 6 (**b**) separate experiments, each performed in technical duplicates. Statistical significance in Ca^{2+} peak responses was determined by a two-tailed student´s t-test. Source data are provided as a Source Data file.

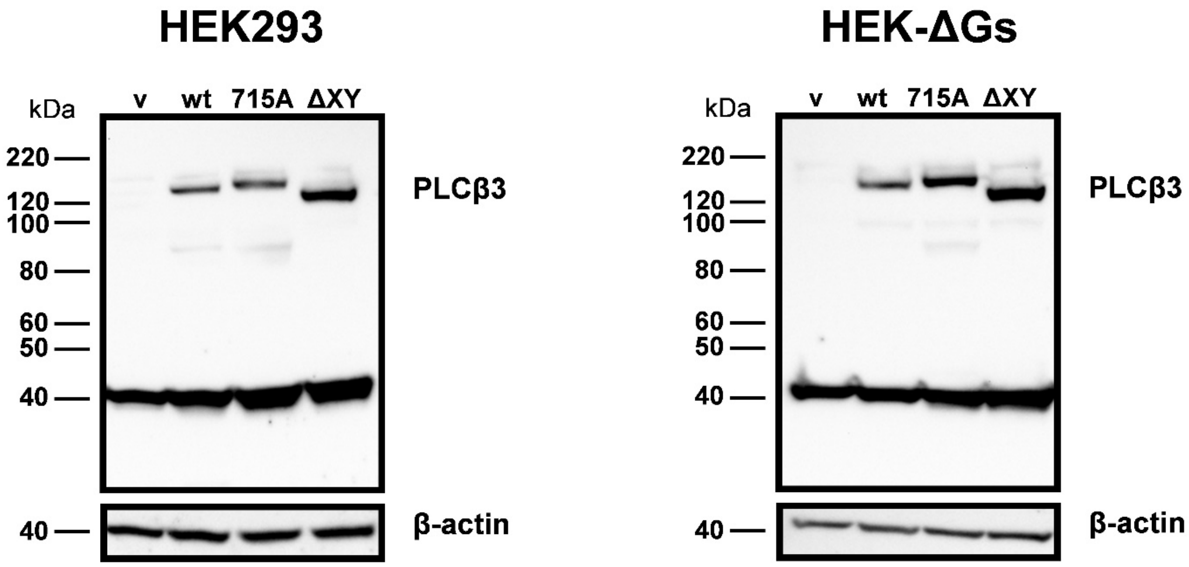

Supplementary Fig. 19: PLCβ3 wild-type and mutant isozymes are expressed at comparable protein abundance in HEK293-wt and HEK-ΔGs cells. Western blot analysis of cell lysates was used to quantify the expression of the indicated PLCβ isozymes in wild-type and Gs-deficient cells. Cell lysates, collected in parallel with the functional experiments, were resolved by SDS-PAGE and immunoblotted with an anti-PLCβ3 mouse monoclonal antibody. β-Actin was used as a loading control. Molecular weight is given in kiloDalton (kDa). Representative blots of n = 3 independent experiments with similar results. Uncropped versions of blots are provided at the end of this Supplementary Information file.

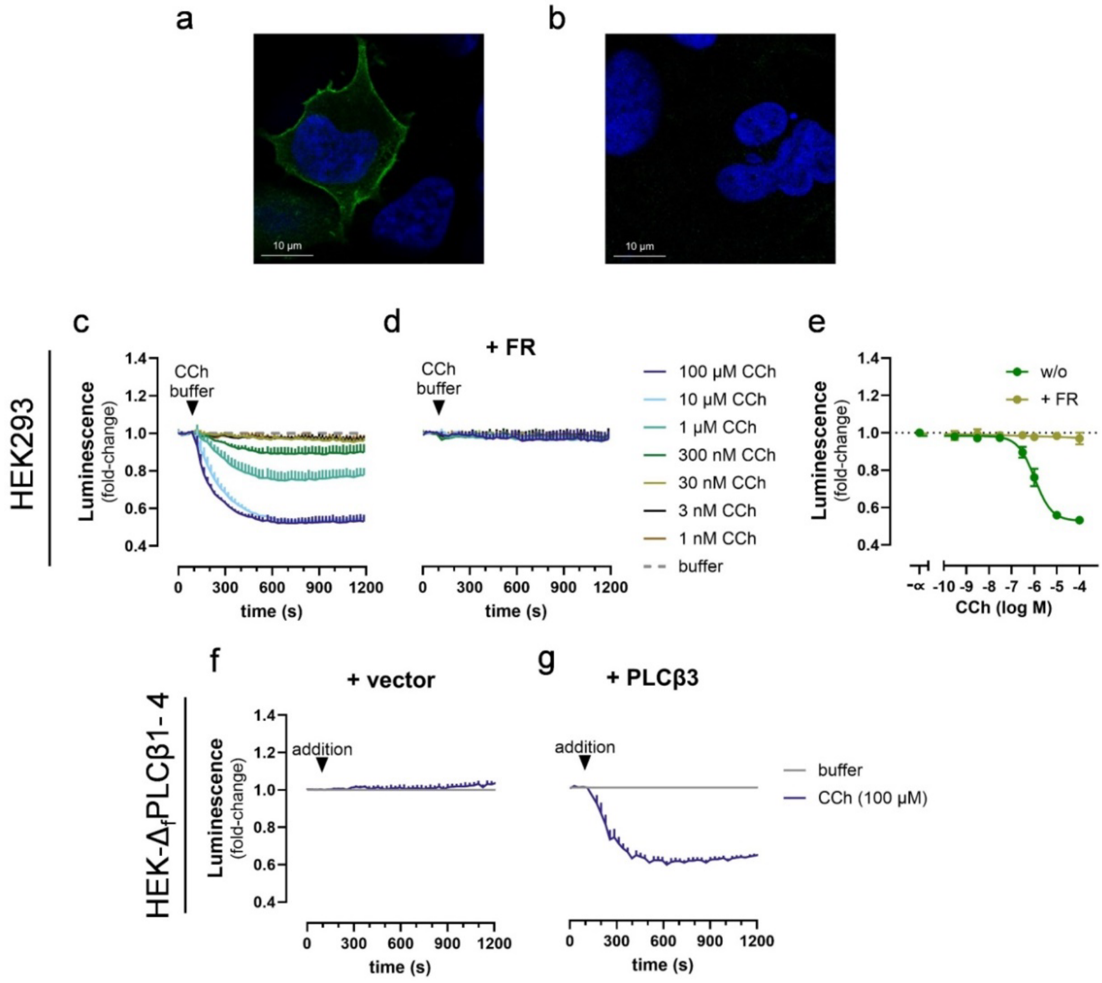

Supplementary Fig. 20: Plasma membrane localization of FLAG-Lg-BiT-CAAX and functional validation of the NanoBiT-based PIP_2 depletion biosensor. a, b Subcellular localization of FLAG-LgBiT-CAAX assessed via confocal microscopy. Confocal images of HEK293A cells transfected to express FLAG-LgBiT-CAAX (**a**) with the same amount of plasmid DNA as in the NanoBiT enzyme complementation experiments or transfected with empty pcDNA3.1 control vector only (**b**). **c**, **d** HEK293A cells transfected to express SmBiT-PLCδ1PH, FLAG-LgBiT-CAAX, and the Gq-coupled muscarinic M_1R were treated with the indicated concentrations of CCh in the absence (**c**) and presence (**d**) of 1 µM FR. Before CCh addition we observed high basal luminescence upon substrate addition, consistent with efficient NanoLuc® complementation between FLAG-LgBiT-CAAX and SmBiT-PLCδ1PH. Stimulation of cells with CCh led to rapid concentration-dependent reduction in luminescence indicative of PIP_2 hydrolysis (**c**). Pretreatment of cells with the Gq/11 inhibitor FR abolished the CCh-induced luminescence decrease, consistent with Gq-dependent and PLCβ-mediated hydrolysis of plasma membrane PIP_2 (**d**). **e** Concentration effect relationship of the data in (**c**) and (**d**). **f, g** Gq-mediated and PLCβ-dependent hydrolysis of plasma membrane PIP_2 is undetectable in PLCβ1-4-deficient HEK293 cells (HEK-Δ_fPLCβ1-4) (**f**) but re-emerges upon PLCβ3 re-expression (**g**). Same experiment as in (**c**) but conceived with HEK-Δ_fPLCβ1-4 cells transfected to re-express PLCβ3 after challenge with vehicle or 100 µM CCh. Real-time recordings show mean + SEM, averaged data are mean + SEM of n = 3 biologically independent experiments, each performed in triplicate. Source data are provided as a Source Data file.

Supplementary References

1. Mukherjee, S. *et al.* A novel biosensor to study cAMP dynamics in cilia and flagella. *eLife* **5** (2016).

2. Gulyás, G. *et al.* Measurement of inositol 1,4,5-trisphosphate in living cells using an improved set of resonance energy transfer-based biosensors. *PloS one* **10,** e0125601 (2015).

Reagents and Commercial Assay Kits

Supplementary Table 1: Reagents

Name	Source	Ref. No.
**5-HT**	Sigma-Aldrich	Cat# H9523-25mg
**Isoproterenol (Iso)**	Sigma Aldrich	Cat# I5627
**A23187**	Sigma-Aldrich	Cat# C7522
**ATP**	Sigma-Aldrich	Cat# A1852-1VL
**Carbachol (CCh)**	Sigma-Aldrich	Cat# C4382-1g
**Coelenterazine**	Carbosynth Limited	Cat# EC14031
**Coelenterazine h**	NanoLight	Cat# 301-1
**Forskolin (Fsk)**	Bachem	Cat# TRC-F701800
**PKi 14-22**	Sigma-Aldrich	Cat# 476485
**Isobutylmethylxanthine (IBMX)**	Sigma-Aldrich	Cat# I5879
**AVP**	Sigma-Aldrich	Cat# 113-79-1
**NECA**	Sigma-Aldrich	Cat# E2387
**PGD_2**	Biomol	Cat# Cay12010-1
**PGE_1**	Sigma-Aldrich	Cat# 745-65-3
**poly-D-lysine**	Sigma-Aldrich	Cat# P2636
**poly-ethylene imine**	Polysciences	Cat# 24313-2
**ICI 118.551**	Sigma Aldrich	Cat# I127
**CGP 20712A**	Sigma Aldrich	Cat# C231
**PKI 14-22 amide**	Sigma Aldrich	Cat# 476485
**HJC0197**	Biolog	Cat# C136
**PTX**	Thermo Fisher Scientific	Cat# PHZ1174
**Gallein**	Sigma-Aldrich	Cat# 2103-64-2
**thapsigargin**	Sigma-Aldrich	Cat# T9033
**2-APB**	Sigma Aldrich	Cat# 524-95-8
**EGF**	Sigma Aldrich	Cat# E9644
**UTP**	Sigma-Aldrich	Cat# U4125

**SNAP-Lumi4-Tb labeling reagent**	Cisbio	Cat# SSNPTBD
**SNAP-Surface® Alexa Fluor® 647**	New England Biolabs	Cat# S9136S
**DMEM-Dulbecco's Modified Eagle Medium**	Thermo Fisher Scientific	Cat# 11965092
**Hanks' buffered salt solution (HBSS)**	Thermo Fisher Scientific	Cat# 14175129
**Penicillin-Streptomycin-Solution**	Thermo Fisher Scientific	Cat# 15140
**Penicillin-Streptomycin-Amphotericin B Solution**	Thermo Fisher Scientific	Cat# 15240
**Fetal Bovine Serum (FBS)**	Sigma Aldrich	Cat# -0804
**4-(2-hydroxyethyl)- 1-piperazineethane-sulfonic acid (HEPES)**	Carl Roth GmbH + Co. KG	Cat# HN77.4
**DH5α Competent Cells**	Thermo Fisher Scientific	Cat# 18265-017
**XL1-Blue Competent Cells**	Stratagene	Cat# 200130

Supplementary Table 2: Commercial Assay Kits

Name	Source	Ref. No.
**ECL Prime Western blotting detection reagent**	GE Healthcare	Cat# RPN2236
**FLIPR® Calcium 5 Assay kit**	Molecular Devices	Cat# R8186
**HTRF-cAMP dynamic 2 kit**	Cisbio International	Cat# 62AM4PEC
**cAMP-Glo™ Assay**	Promega Corporation	Cat# V1501
**Pierce BCA Protein Assay**	Thermo Fisher Scientific	Cat# 23225

Uncropped scans of blots in Supplementary Fig. 19

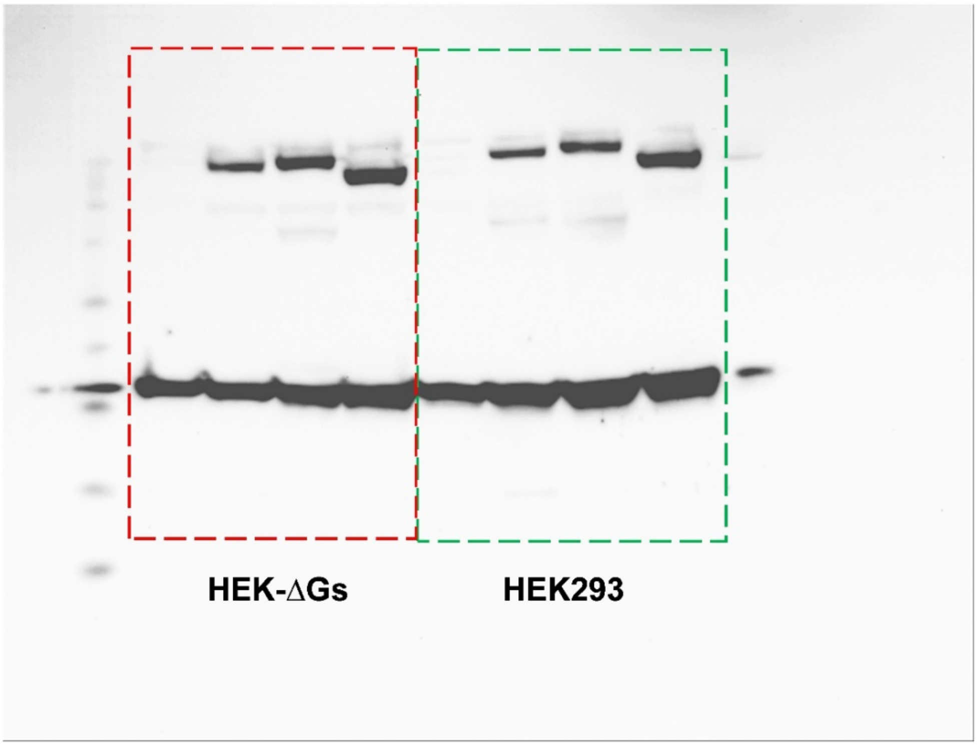

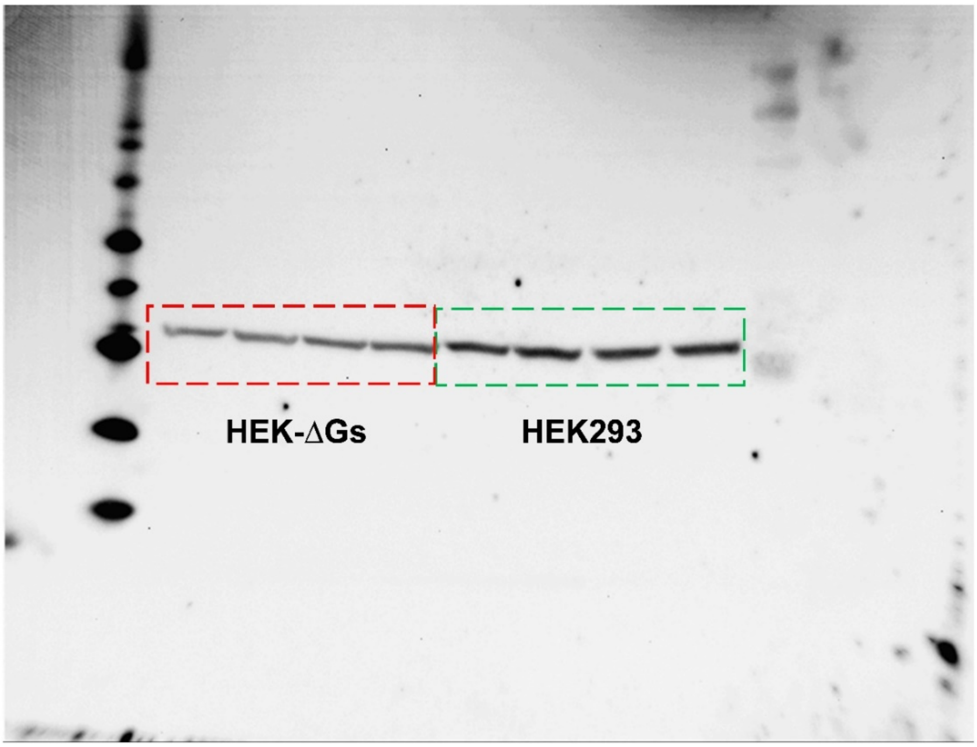

Supplement: Supplementary file 1 — Supplementary Information [file 41467_2024_51991_MOESM1_ESM.pdf]
