# Supplementary material for: A molecular mechanism to diversify Ca2+ signaling downstream of Gs protein-coupled receptors
Source: Nat Commun. 2024 Sep 3;15:7684. doi: 10.1038/s41467-024-51991-6 (PMC11372221; doi:10.1038/s41467-024-51991-6)
Supplement: Supplementary file 3 — Reporting Summary [file 41467_2024_51991_MOESM3_ESM.pdf]

nature portfolio

Corresponding author(s): Evi Kostenis

Last updated by author(s): Aug 7, 2024

Reporting Summary

Nature Portfolio wishes to improve the reproducibility of the work that we publish. This form provides structure for consistency and transparency in reporting. For further information on Nature Portfolio policies, see our Editorial Policies and the Editorial Policy Checklist.

Statistics

For all statistical analyses, confirm that the following items are present in the figure legend, table legend, main text, or Methods section.

n/a	Confirmed	
☐	☒	The exact sample size (*n*) for each experimental group/condition, given as a discrete number and unit of measurement
☐	☒	A statement on whether measurements were taken from distinct samples or whether the same sample was measured repeatedly
☐	☒	The statistical test(s) used AND whether they are one- or two-sided<br>*Only common tests should be described solely by name; describe more complex techniques in the Methods section.*
☒	☐	A description of all covariates tested
☐	☒	A description of any assumptions or corrections, such as tests of normality and adjustment for multiple comparisons
☐	☒	A full description of the statistical parameters including central tendency (e.g. means) or other basic estimates (e.g. regression coefficient) AND variation (e.g. standard deviation) or associated estimates of uncertainty (e.g. confidence intervals)
☐	☒	For null hypothesis testing, the test statistic (e.g. *F*, *t*, *r*) with confidence intervals, effect sizes, degrees of freedom and *P* value noted<br>*Give P values as exact values whenever suitable.*
☒	☐	For Bayesian analysis, information on the choice of priors and Markov chain Monte Carlo settings
☒	☐	For hierarchical and complex designs, identification of the appropriate level for tests and full reporting of outcomes
☒	☐	Estimates of effect sizes (e.g. Cohen's *d*, Pearson's *r*), indicating how they were calculated

Our web collection on statistics for biologists contains articles on many of the points above.

Software and code

Policy information about availability of computer code

Data collection	Data were generated on the following devices: FlexStation® 3 Multimode Bench Top reader (Molecular Devices, San Jose, CA, USA), Mithras LB 940 multimode plate reader (Berthold Technologies, Bad Wildbad, Germany), PHERAstar FSX microplate reader (BMG labtech, Ortenberg, Germany), CLARIOstar Plus multimode plate reader (BMG labtech, Ortenberg, Germany), Zeiss Axio Observer Z1 (Carl Zeiss Gmbg, Oberkochen, Deutschland), Spark multi-mode plate reader (TECAN, Männedorf, Switzerland) and Corning® EPIC® biosensor (Corning, NY, USA).<br>Data collection was performed with Microsoft Excel 2019.
Data analysis	Data and statistical analyses were performed using GraphPad Prism version: 10.2.3

For manuscripts utilizing custom algorithms or software that are central to the research but not yet described in published literature, software must be made available to editors and reviewers. We strongly encourage code deposition in a community repository (e.g. GitHub). See the Nature Portfolio guidelines for submitting code & software for further information.

Data

Policy information about availability of data

All manuscripts must include a data availability statement. This statement should provide the following information, where applicable:

- Accession codes, unique identifiers, or web links for publicly available datasets
- A description of any restrictions on data availability
- For clinical datasets or third party data, please ensure that the statement adheres to our policy

All data generated and analyzed during this study are included in this published article and the Supplementary Information. Additional data related to this paper may be requested from the authors.

Research involving human participants, their data, or biological material

Policy information about studies with human participants or human data. See also policy information about sex, gender (identity/presentation), and sexual orientation and race, ethnicity and racism.

Reporting on sex and gender	No human research participants were included in this study.
Reporting on race, ethnicity, or other socially relevant groupings	n/a
Population characteristics	n/a
Recruitment	n/a
Ethics oversight	n/a

Note that full information on the approval of the study protocol must also be provided in the manuscript.

Field-specific reporting

Please select the one below that is the best fit for your research. If you are not sure, read the appropriate sections before making your selection.

☒ Life sciences ☐ Behavioural & social sciences ☐ Ecological, evolutionary & environmental sciences

For a reference copy of the document with all sections, see nature.com/documents/nr-reporting-summary-flat.pdf

Life sciences study design

All studies must disclose on these points even when the disclosure is negative.

Sample size	Sample size was chosen based on prior experience of the investigators with similar experiments previously published. The authors have published numerous peer-reviewed papers demonstrating clear positive findings with similar sample sizes for the types of experiments included (e.g., PMID: 29362459, 35087057, 36402762, 33202251).
Data exclusions	No data were excluded
Replication	All experimental findings were reproduced in several independent experiments, as indicated in the figure legends.
Randomization	Randomization was not relevant to our study as it was based on cellular experiments and aimed to test the effect of different agonist on the same sample. Our study was not a clinical trial that is dependent of randomization. Experiments were performed using cells and all variables could be controlled.
Blinding	Blinding was not relevant because there were no subject differences for the different treatments as well as transfection of the cells was conducted by the same investigators.

Reporting for specific materials, systems and methods

We require information from authors about some types of materials, experimental systems and methods used in many studies. Here, indicate whether each material, system or method listed is relevant to your study. If you are not sure if a list item applies to your research, read the appropriate section before selecting a response.

Materials & experimental systems

n/a	Involved in the study	
	x	Antibodies
	x	Eukaryotic cell lines
x		Palaeontology and archaeology
	x	Animals and other organisms
x		Clinical data
x		Dual use research of concern
x		Plants

Methods

n/a	Involved in the study	
x		ChIP-seq
x		Flow cytometry
x		MRI-based neuroimaging

Antibodies

Antibodies used	All antibodies used are described in the Methods section.<br>Anti-PLCβ3 mouse monoclonal antibody; dilution1:500; Santa Cruz Biotechnology; Cat.: sc-133231.<br>Anti-mouse IgG goat antibody HRP; dilution 1:20,000; Sigma; Cat.: A4416.<br>Anti-β-actin rabbit antibody; dilution1:10,000; BioLegend; Cat.: 622102.<br>Anti-rabbit IgG goat antibody HRP; dilution: 1:20,000; ABIN; Cat.: 102010.<br>Anti-FLAG M2 antibody (Sigma Aldrich, #F1804, 1:1000). Polyclonal goat anti-mouse Alexa Fluor 488-conjugated antibody (Invitrogen, #A28175, 1:1000).
Validation	Validation for the species (human) and the application was performed by the manufacturer using fluorescence microscopy and Western Blot analysis. For details, please visit the manufacturers´ web sites.

Eukaryotic cell lines

Policy information about cell lines and Sex and Gender in Research

Cell line source(s)	HEK293 cells (source: ThermoFisher). HEK293A cells (source: ThermoFisher). HEK293-T cells (source: Jesper M. Mathiesen). ΔGsHEK293 cells (source: Asuka Inoue). ΔAC3/6 Hek293 cells (source: Val J. Watts). HEK-ΔfPLCβ1-4 cells (source: Asuka Inoue).<br>Details and citations on cell generation and characterization are given in the Methods section.
Authentication	Cells were not further authenticated by the authors
Mycoplasma contamination	All cell lines were routinely screened for possible mycoplasma contamination. Results were always negative.
Commonly misidentified lines<br>(See ICLAC register)	No commonly misidentified cell lines were used in the study,

Animals and other research organisms

Policy information about studies involving animals; ARRIVE guidelines recommended for reporting animal research, and Sex and Gender in Research

Laboratory animals	Embryos and neonatal mice, C57Bl6/J background, Janvier Labs (France).
Wild animals	No wild animals were used in this study.
Reporting on sex	Sex was not considered in the study.
Field-collected samples	No field collected samples were used in this study.
Ethics oversight	All animal experiments were performed in agreement with the German law of animal protection and local institutional animal care committees (Landesamt für Natur, Umwelt und Verbraucherschutz, LANUV). Mice were raised under a normal circadian light/dark cycle of each 12 h and animals were given water and complete diet (ssniff Spezialdiäten) ad libitum (approved by the Veterinäramt Bonn, §11). According to the German animal protection law (Tierschutzgesetz) §4 paragraph 3, animals can be sacrificed for scientific purposes / interests for harvesting tissue or to isolate cells. Consequently, special approval for harvesting embryonic and neonatal cells was not required.

Note that full information on the approval of the study protocol must also be provided in the manuscript.

Plants

Seed stocks	*Report on the source of all seed stocks or other plant material used. If applicable, state the seed stock centre and catalogue number. If plant specimens were collected from the field, describe the collection location, date and sampling procedures.*
Novel plant genotypes	*Describe the methods by which all novel plant genotypes were produced. This includes those generated by transgenic approaches, gene editing, chemical/radiation-based mutagenesis and hybridization. For transgenic lines, describe the transformation method, the number of independent lines analyzed and the generation upon which experiments were performed. For gene-edited lines, describe the editor used, the endogenous sequence targeted for editing, the targeting guide RNA sequence (if applicable) and how the editor was applied.*
Authentication	*Describe any authentication procedures for each seed stock used or novel genotype generated. Describe any experiments used to assess the effect of a mutation and, where applicable, how potential secondary effects (e.g. second site T-DNA insertions, mosiacism, off-target gene editing) were examined.*
